# Alterations of the Nerves in Chronic Rheumatism

**Published:** 1882-03

**Authors:** 


### Alterations of the Nerves in Chronic Rheumatism.

MM. Leloir and Degerine reported to the Societe de Biologie, April 2 (abst. in *Le Progres Medical*), that in case of chronic rheumatism, with considerable muscular atrophy and rapid eschars, they found the cutaneous nerves adjacent to the eschars affected with atrophic parenchymatous neuritis. They thought that the alteration in the nerves was anterior to the eschars, and saw evidence of this in the rapidity of the ulceration itself. The histological examination of the cord remained yet to be made.

